# Supplementary material for: Human iPSC-derived mature microglia retain their identity and functionally integrate in the chimeric mouse brain
Source: Nat Commun. 2020 Mar 27;11:1577. doi: 10.1038/s41467-020-15411-9 (PMC7101330; doi:10.1038/s41467-020-15411-9)
Supplement: Supplementary file 1 — Supplementary information [file 41467_2020_15411_MOESM1_ESM.pdf]

Supplementary Information

Human iPSC-derived mature microglia retain their identity and functionally integrate in the chimeric mouse brain

Xu et al.

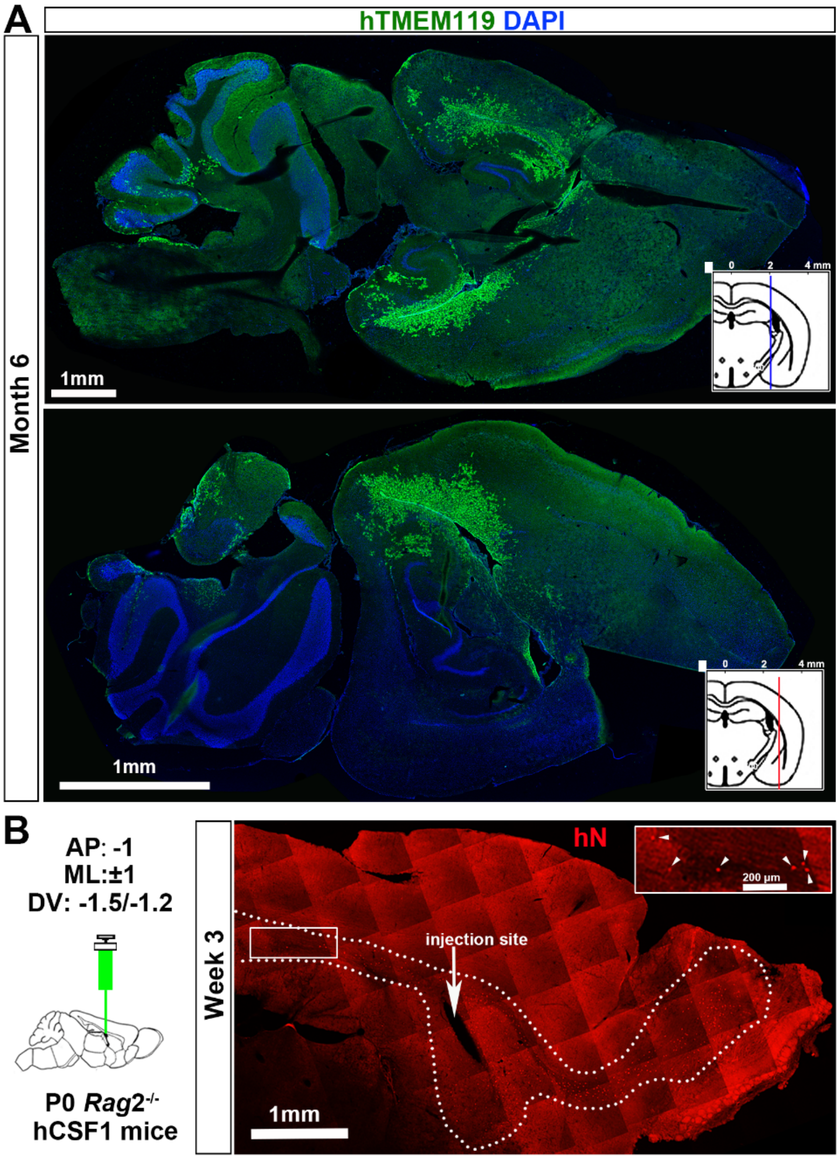
A
hTMEM119 DAPI
Month 6
1mm
0 2 4 mm
1mm
0 2 4 mm
B
AP: -1
ML:±1
DV: -1.5/-1.2
P0 Rag2-/-
hCSF1 mice
Week 3
hN
200 µm
injection site
1mm

1

Supplementary Figure 1. Distribution of hPSC-derived microglia in the mouse brain. (A) Representative images from sagittal brain sections showing the distribution of xenografted hPSC-derived microglia at six months post-transplantation. Anti-human-specific TMEM119 (hTMEM119) staining selectively labels xenografted hPSC-derived microglia. Scale bar: 1 mm.
(B) A schematic diagram and representative images from a sagittal brain section showing the distribution of xenografted hPSC-derived microglia at three weeks post-transplantation. Human PSC-derived PMPs were transplanted into the lateral ventricles. Anti-human nuclei (hN) staining selectively labels xenografted hPSC-derived donor cells. Arrow indicates the injection site. Arrowheads indicate hN^+ cells. Scale bar: 1 mm or 200 μm in the original or enlarged images, respectively.

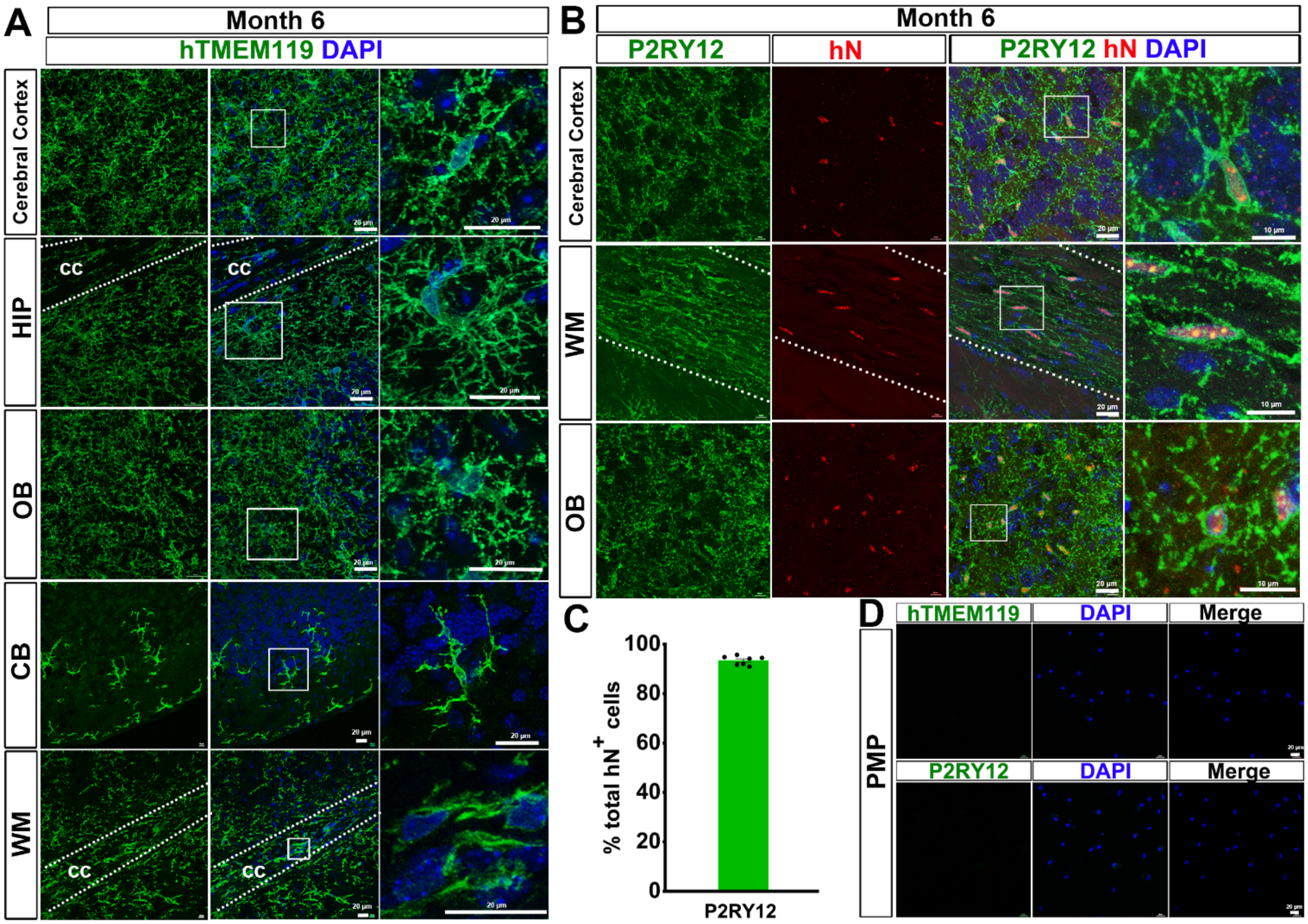
A
Month 6
hTMEM119 DAPI
Cerebral Cortex
HIP
OB
CB
WM
CC
B
Month 6
P2RY12
hN
P2RY12 hN DAPI
Cerebral Cortex
WM
OB
C
% total hN+ cells
100
80
60
40
20
0
P2RY12
D
hTMEM119
DAPI
Merge
PMP
P2RY12
DAPI
Merge

1

Supplementary Figure 2. Identification of hPSC-derived microglia in the mouse brain.

(A) Representative images showing the morphology of $hTMEM119^+$ hPSC-derived microglia in the cerebral cortex, hippocampus (HIP), olfactory bulb (OB), cerebellum (CB) and white matter (WM) at 6 months post-transplantation. CC: corpus callosum. Scale bars: 50 μm or 20 μm in the original or enlarged images, respectively.

(B) Representative images of P2RY12- and hN-expressing cells in the cerebral cortex, WM and OB at 6 months post-transplantation. Scale bars, 20 μm or 10 μm in the original or enlarged images, respectively.

(C) Quantification of the percentage of $P2RY12^+$ cells in total hN^+ cells (n = 7 mice). The data are pooled from the mice that received transplantation of microglia derived from both hESCs and hiPSCs. Data are presented as mean ± s.e.m.

(D) Representative images showing no expression of hTMEM119 and P2RY12 in the hPSC-derived PMPs. Scale bars, 20 μm.

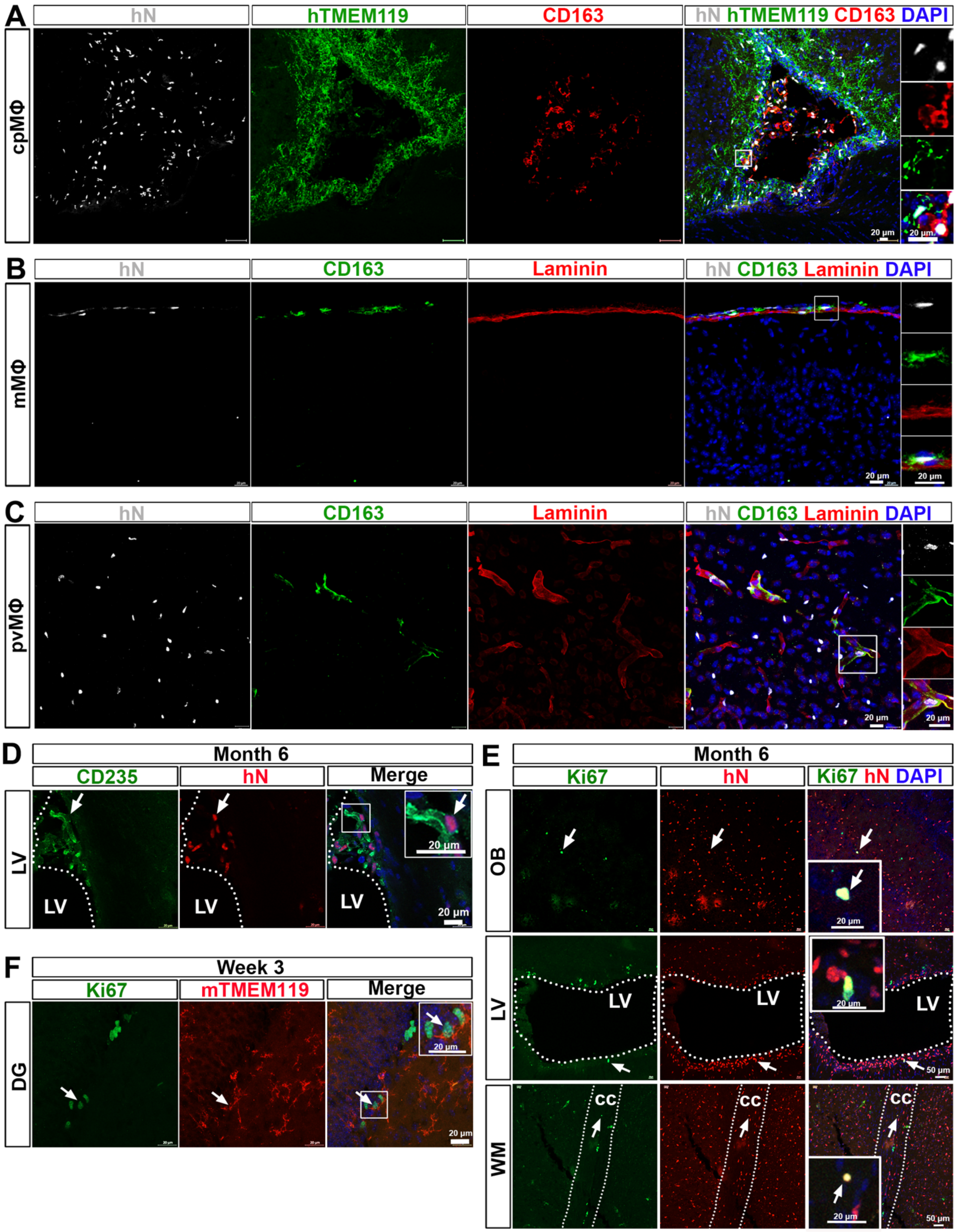
A
hN
hTMEM119
CD163
hN hTMEM119 CD163 DAPI
cpMΦ
20 μm
B
hN
CD163
Laminin
hN CD163 Laminin DAPI
mMΦ
20 μm
C
hN
CD163
Laminin
hN CD163 Laminin DAPI
pvMΦ
20 μm
D
Month 6
CD235
hN
Merge
LV
20 μm
E
Month 6
Ki67
hN
Ki67 hN DAPI
OB
LV
WM
cc
50 μm
20 μm
F
Week 3
Ki67
mTMEM119
Merge
DG
20 μm

1

Supplementary Figure 3. Characterization of donor-derived CNS macrophages in the mouse brain.

(A) Representative images of $hTMEM119^-/hN^+/CD163^+$ choroid plexus macrophage (cpMΦ) in choroid plexus at 6 months post-transplantation. Scale bars, 20 μm in the original or enlarged images.

(B) Representative images of hN^+ and $CD163^+$ meningeal macrophage (mMΦ) around the $laminin^+$ meninges at 6 months post-transplantation. Scale bars, 20 μm in the original or enlarged images.

(C) Representative images of hN^+ and $CD163^+$ perivascular macrophages (pvMΦ) in perivascular spaces at 6 months post-transplantation. Scale bars, 20 μm in the original or enlarged images.

(D) Representative images of CD235- and hN-expressing cells in the lateral walls of lateral ventricle (LV) at 6 months post-transplantation. Scale bars, 20 μm in the original or enlarged images.

(E) Representative images of Ki67- and hN-expressing cells in the olfactory bulb (OB), lateral walls of LV, and white matter (WM) at 6 months post-transplantation. cc, corpus callosum. Scale bars, 50 μm or 20 μm in the original or enlarged images, respectively.

(F) Representative images of Ki67- and mTMEM119-expressing cells in the dentate gyrus (DG) at 3 weeks chimeric mice. Scale bars, 20 μm in the original or enlarged images.

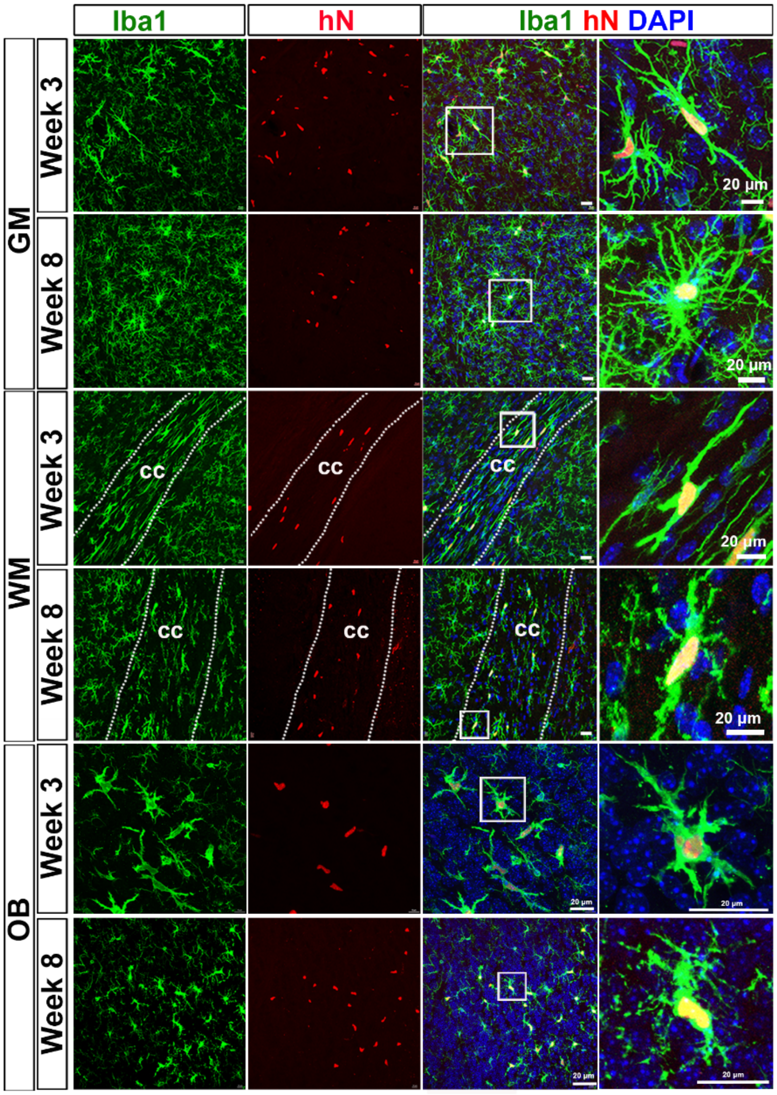

Iba1
hN
Iba1 hN DAPI
GM
Week 3
Week 8
WM
Week 3
Week 8
OB
Week 3
Week 8
cc
20 μm

1

Supplementary Figure 4. Characterization of Human PSC-derived microglia in the mouse brain. Representative images of $Iba1^+/hN^+$ hPSC-derived microglia in the grey matter (GM), white matter (WM) and olfactory bulb (OB) at 3 weeks and 8 weeks post-transplantation. cc: corpus callosum. Scale bars, 20 μm in both the original and enlarged images.

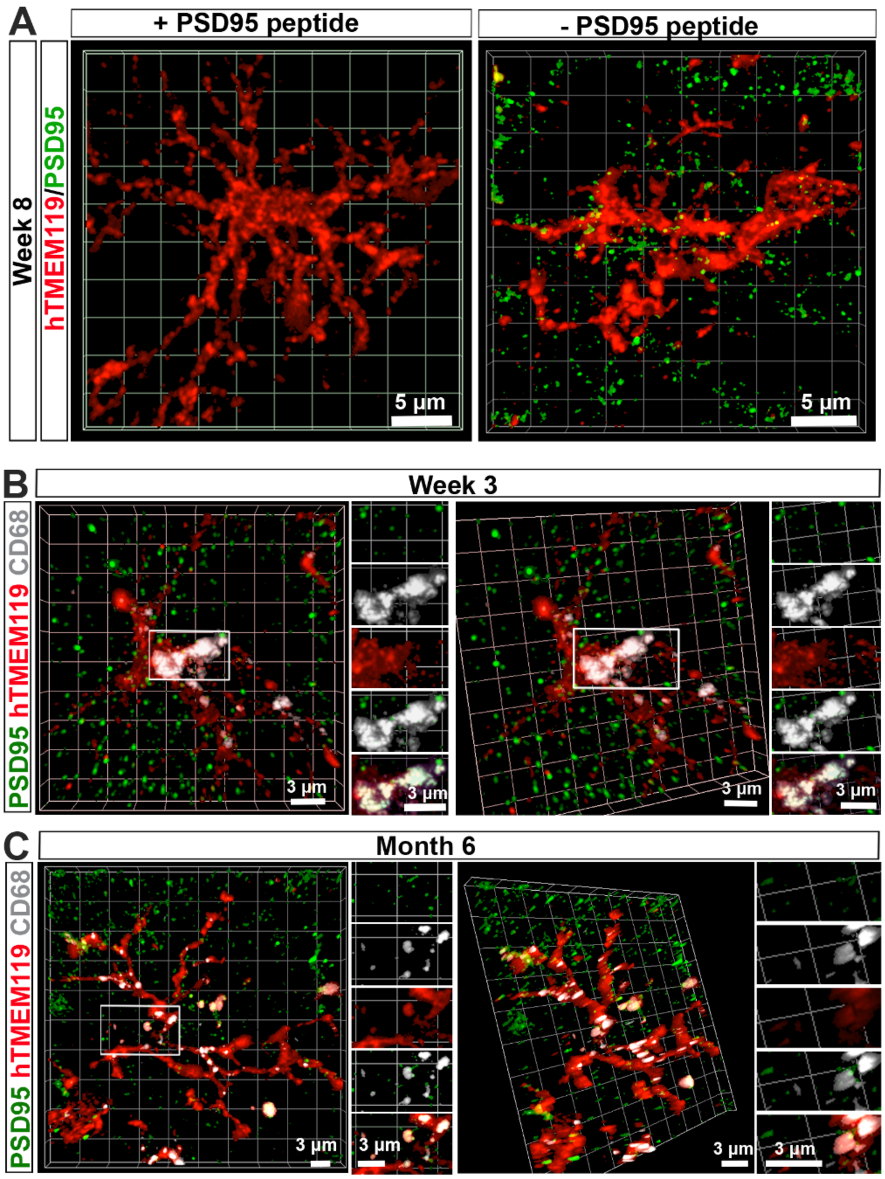
A
+ PSD95 peptide
- PSD95 peptide
Week 8
hTMEM119/PSD95
5 µm
5 µm
B
Week 3
PSD95 hTMEM119 CD68
3 µm
3 µm
3 µm
3 µm
C
Month 6
PSD95 hTMEM119 CD68
3 µm
3 µm
3 µm
3 µm

1

Supplementary Figure 5. Characterization of synaptic pruning by hPSC-derived microglia in the chimeric mouse brain.

(A) Representative images showing PSD95 staining after incubation with PSD95 peptide (left) or without PSD95 peptide (right). Scale bars, 5μm.

(B and C) Representative 3D reconstruction of super-resolution images showing the colocalization of hTMEM119, PSD95, and CD68 staining in grey matter at 3 weeks (B) and 6 months (C) post-transplantation. Scale bars, 3 μm in the original and enlarged images.

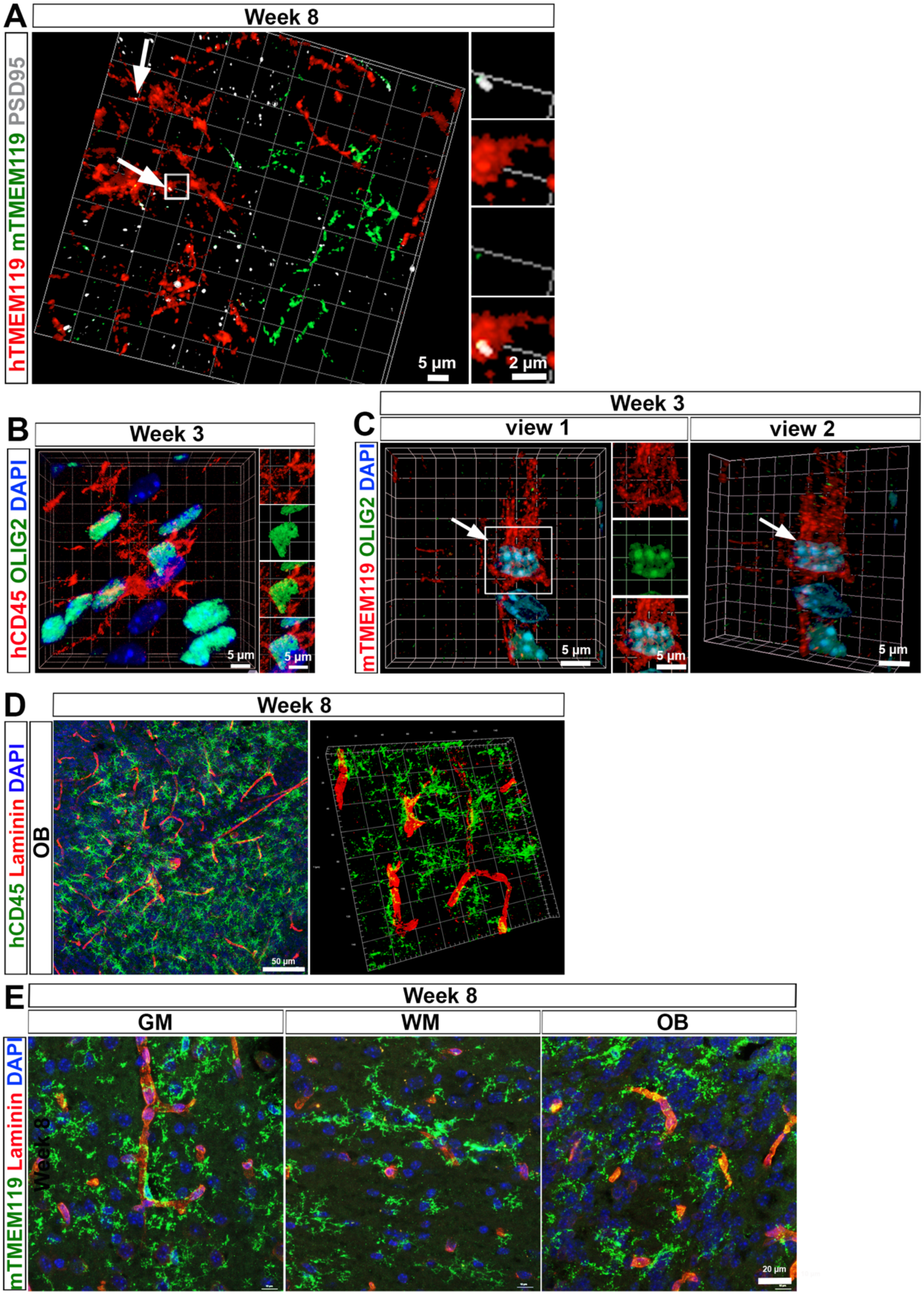
A
Week 8
hTMEM119 mTMEM119 PSD95
5 µm
2 µm
B
Week 3
hCD45 OLIG2 DAPI
5 µm
5 µm
C
Week 3
view 1
view 2
mTMEM119 OLIG2 DAPI
5 µm
5 µm
5 µm
D
Week 8
hCD45 Laminin DAPI
OB
50 µm
E
Week 8
GM
WM
OB
mTMEM119 Laminin DAPI
Week 8
20 µm

1

Supplementary Figure 6. Functional comparison between human PSC-derived microglia and mouse host microglia in the chimeric mouse brain.

(A) Representative 3D reconstruction images showing hTMEM119$^+$ donor-derived microglia engulf PSD95$^+$ puncta in grey matter at 8 weeks post-transplantation. Very few mTMEM119$^+$ mouse host microglia interact with PSD95. Scale bars, 5 μm or 2 μm in the original and enlarged images.

(B) Representative 3D reconstruction images showing that hCD45$^+$ hPSC-derived microglia phagocytize OLIG2$^+$ oligodendroglial cells in the corpus callosum at 3 weeks post-transplantation. Scale bars, 5 μm.

(C) Representative 3D reconstruction of super-resolution images showing that mTMEM119$^+$ mouse host microglia phagocytize OLIG2$^+$ oligodendroglial cells in the corpus callosum in 3 weeks old chimeric mice. Scale bars, 5 μm.

(D) Representative images showing the interaction between laminin$^+$ blood vessel and hCD4$^+$ human microglia in the olfactory bulb (OB) and white matter at 8 weeks post-transplantation. Scale bars, 50 μm.

(E) Representative images showing interaction between laminin$^+$ blood vessel and mTMEME119$^+$ mouse host microglia in grey matter (GM), white matter (WM) and OB at 8 weeks post-transplantation. Scale bars, 20 μm.

A

Sample	Estimated Number of Cells	Mean Reads per Cell	Number of Reads	Valid Barcodes	Reads Mapped to Genome	hg19 Reads Mapped to Genome	mm10 Reads Mapped to Genome	Reads Mapped Confidently to Genome	hg19 Reads Mapped Confidently to Genome	mm10 Reads Mapped Confidently to Genome
s1	6,390	14,469	92,458,639	96.80%	94.00%	7.20%	86.90%	82.00%	6.70%	75.30%
s2	8,811	10,803	95,192,063	96.70%	93.40%	7.40%	86.20%	81.80%	6.90%	74.90%
s3	6,742	15,020	101,270,517	96.50%	93.70%	14.40%	79.50%	87.30%	13.60%	73.80%
s4	8,031	12,957	104,060,861	96.60%	92.20%	4.50%	87.90%	81.80%	4.10%	77.70%
**Mean**	**7,494**	**13,312**	**98,245,520**	**96.65%**	**93.33%**	**8.38%**	**85.13%**	**83.23%**	**7.83%**	**75.43%**

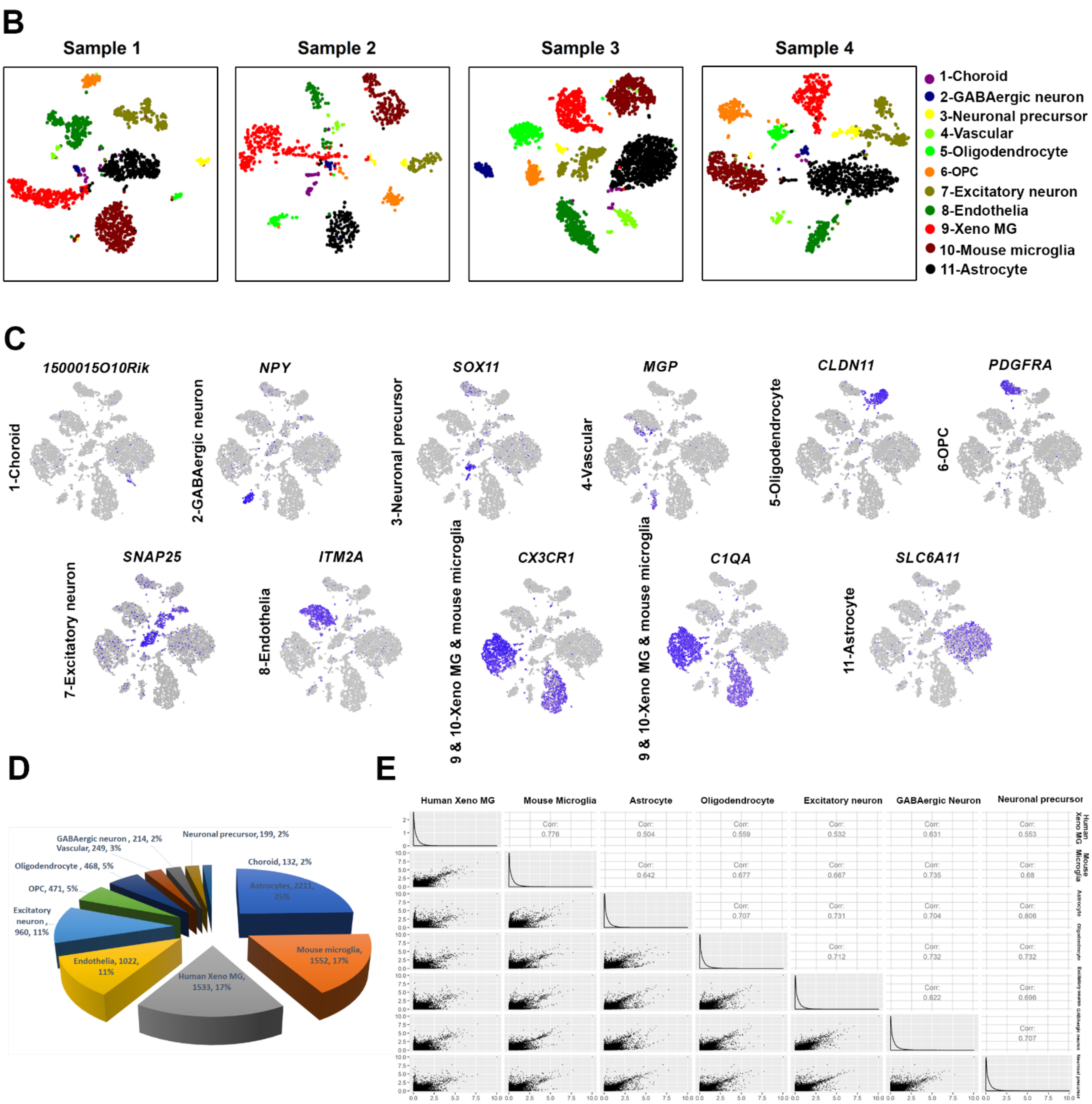

Supplementary Figure 7. Single-cell RNA-sequencing of hiPSC microglial chimeric mouse brains.

(A) A table summarizing the observed numbers of single-cell RNA-sequencing reads and those matching human (hg19) or mouse (mm10) genome with high confidence. The numbers (with percentages in parentheses) of detected mouse/human microglia, respectively, detected in each dissected sample were: Sample 1: 442/395 (21.6%/19.3%); Sample 2: 176/274 (18.0%/28.1%); Sample 3: 551/537 (14.6%/14.3%); Sample 4: 383/327 (17.2%/14.7%).

(B) tSNE plots showing that the 11 cell type clusters are individually reproduced in each of the four sampled 6-month-old chimeric mouse brains. For this analysis, results from each replicate animal were analyzed and clustered separately.

(C) tSNE plots showing the expression of representative genes in each of the 11 clusters. Blue color saturation indicates relative log normalized counts in each cell (dot, as identified by unique barcode).

(D) A pie chart summarizing the numbers of cells (barcodes) identified within each clustered cell type.

(E) Scatter plot array showing the pairwise correlation of average expression for each cell type. Notably, the Xeno MG and mouse microglia clusters had the highest correlation coefficient value, 0.776.

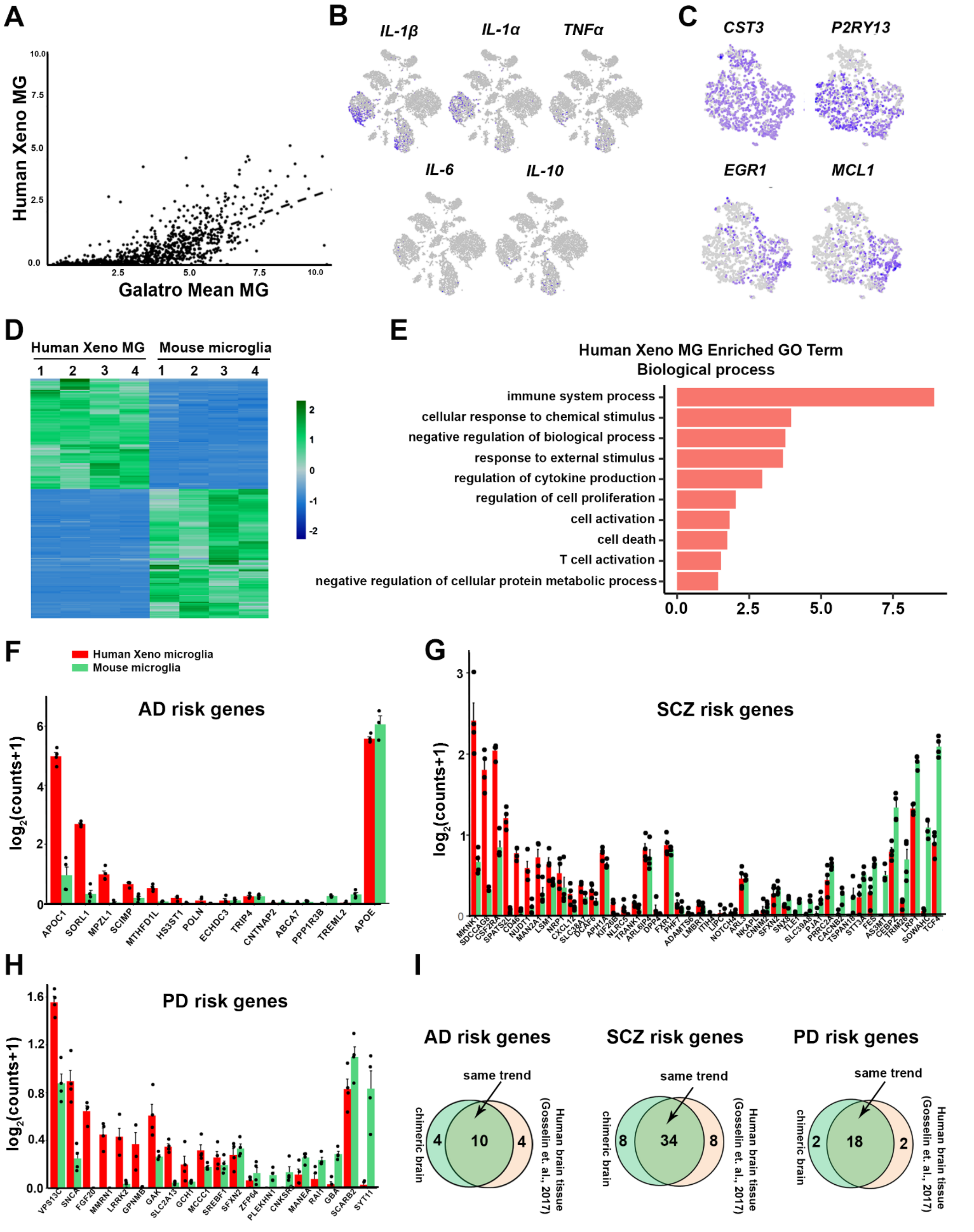
A
Human Xeno MG
Galatro Mean MG
0.0
2.5
5.0
7.5
10.0
B
IL-1β
IL-1α
TNFα
IL-6
IL-10
C
CST3
P2RY13
EGR1
MCL1
D
Human Xeno MG
Mouse microglia
1 2 3 4 1 2 3 4
2
1
0
-1
-2
E
Human Xeno MG Enriched GO Term
Biological process
immune system process
cellular response to chemical stimulus
negative regulation of biological process
response to external stimulus
regulation of cytokine production
regulation of cell proliferation
cell activation
cell death
T cell activation
negative regulation of cellular protein metabolic process
0.0
2.5
5.0
7.5
F
Human Xeno microglia
Mouse microglia
AD risk genes
log2(counts+1)
APOC1
SORL1
MPZL1
SCIMP
MTHFD1L
HS3ST1
POLN
ECHDC3
TRIP4
CNTNAP2
ABCA7
PPP1R3B
TREML2
APOE
G
SCZ risk genes
log2(counts+1)
H
PD risk genes
log2(counts+1)
VPS13C
SNCA
FGF20
MMRN1
LRRK2
GPNMB
GAK
SLC2A13
GCH1
MCCC1
SREBF1
SFXN2
ZFP64
PLEKHN1
CNKSR3
MANEA
RAI1
GBA
SCARB2
SYT11
I
AD risk genes
SCZ risk genes
PD risk genes
same trend
chimeric brain
Human brain tissue
(Gosselin et. al., 2017)
4
10
4
8
34
8
2
18
2

Supplementary Figure 8. Transcriptomic profile of Xeno MG developed in the mouse brain. (A) Scatter plot showing correlation of human Xeno MG with human adult microglia dataset reported in Galatro et. al., 2017. Expression levels are normalized reads per million (RPM). The 10X Genomics data from the Human Xeno MG matched only the relatively higher levels of expression ($\log_2$ RPM > 2) of the deeper (more RNAseq reads per sample) dataset from Galatro. However, transcripts above this threshold exhibited a strong correlation ($r^2 = 0.3851$, $p < 2.2 \times 10^{-16}$, ANOVA comparing linear model of regression with a null model).
(B) t-SNE plots for mRNA expression of acute pro-inflammatory cytokines, *IL-1β, IL-1α,* and *TNF-α,* and chronic pro-inflammatory cytokine, *IL-6* and anti-inflammatory cytokine, *IL-10*.
(C) t-SNE plots for *CST3, P2RY13, EGR1,* and *MCL1* mRNA expression (log normalized counts) within the selected human Xeno MG cluster.
(D) A heatmap showing the DEGs in individual samples (n = 4 mice) of human and mouse microglia from 6-month old chimeric mouse brains. Expression levels (log normalized counts) were normalized to mean expression of all samples, producing a Z-score, with color assignments indicated by the legend.
(E) Enriched gene ontology (GO) biological process terms for the upregulated DEGs in human Xeno MG, plotted as the -log10 (p-value) of enrichment.
(F-H) Bar plots showing the expression (mean ± SEM, n = 4 mice) of Alzheimer's disease (AD), schizophrenia (SCZ), or Parkinson's disease (PD)-associated genes in Xeno MG and mouse microglia. All these genes were reported to be differentially expressed between human and mouse microglia in Gosselin et al., 2017.
(I) Venn diagram showing that the majority of genes that were reported to be differentially expressed between human and mouse microglia are recapitulated in our chimeric mouse model (10 of 14 for AD, 34 of 42 for SCZ, and 18 of 20 for PD).

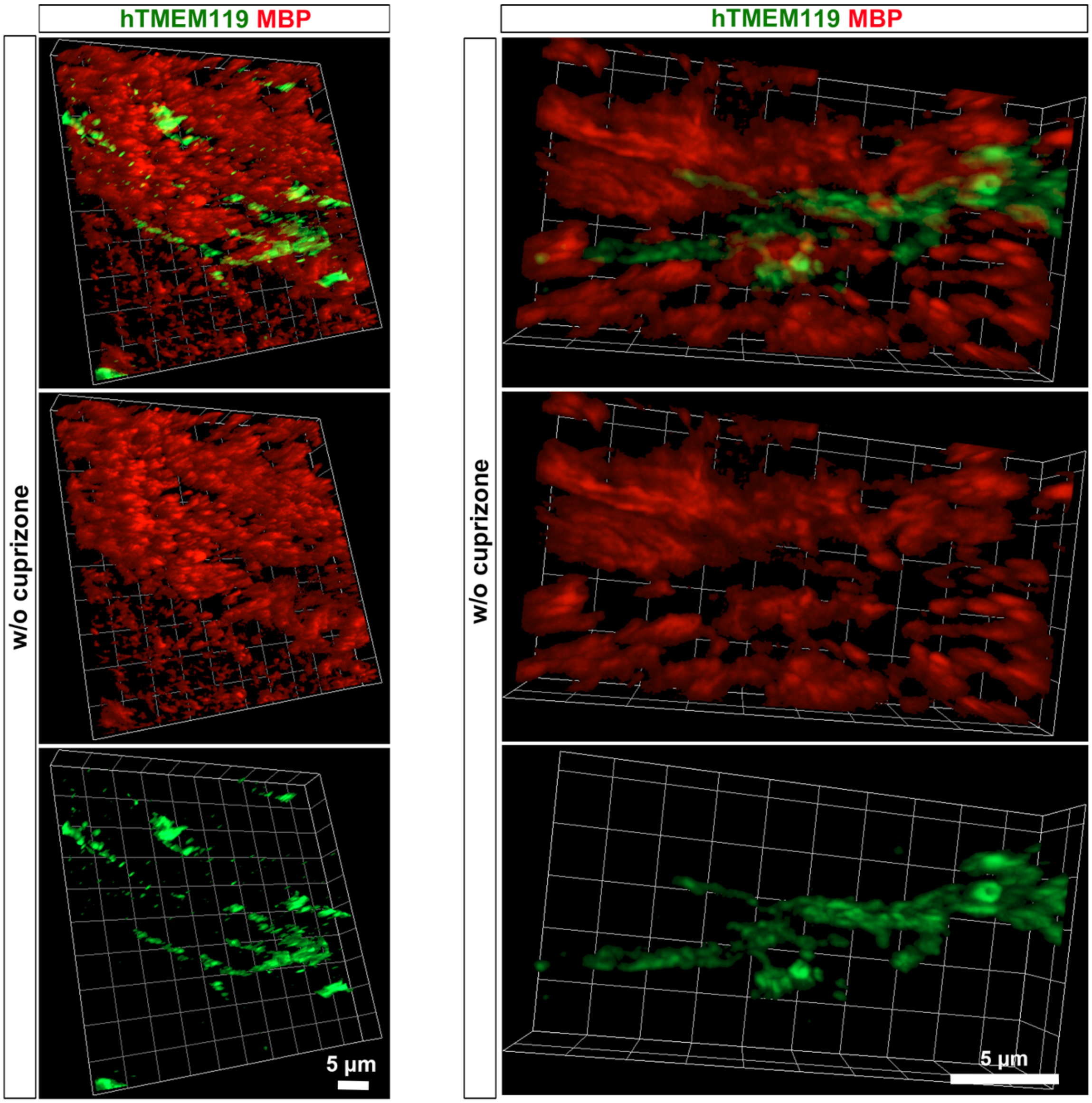
hTMEM119 MBP
w/o cuprizone
5 μm
hTMEM119 MBP
w/o cuprizone
5 μm

1

Supplementary Figure 9. Representative 3D reconstruction of super-resolution images showing hTMEM119$^+$ donor-derived microglia and MBP$^+$ myelin structure in the corpus callosum at after 4 weeks of control diet without cuprizone. Scale bars, 5 μm.

Supplementary Data 1. The top 10 enriched genes in each cluster. For each cell type cluster identified by Seurat analysis, the top ten genes distinguishing that cluster from all other cells are shown, as calculated by the FindAllMarkers function. The p-values (p_val) were calculated using a Wilcoxon Rank Sum test, two-tailed, and were adjusted for multiple measurements using Benjamini-Hochberg (p_val_adj).

Supplementary Data 2. A list of differentially expressed genes between Xeno MG and mouse microglia. Gene expression differences between human and mouse microglial clusters were calculated by the Seurat FindMarkers function. The p-values (p_val) were calculated using a Wilcoxon Rank Sum test, two-tailed, and were adjusted for multiple measurements using Benjamini-Hochberg (p_val_adj).

Supplementary Table 1. A list of antibodies used.

Antibodies	Vendor/Catalog #.	Type	Dilution
CD163	R&D system / AF1067	Goat IgG	1:200
CD235	Thermo Fisher Scientific / PA5-27154	Rabbit IgG	1:100
CD43	Thermo Fisher Scientific / 14-0439-82	Mouse IgG1	1:25
CD45	Thermo Fisher Scientific / 14-9457-82	Mouse IgG1	1:40
CD68	Invitrogen / MA5-13324	Mouse IgG1	1:100
CD74	Invitrogen / JF40-10	Rabbit IgG	1:200
hTMEM119	Thermo Fisher Scientific / PA-62505	Rabbit IgG	1:400
mTMEM119	Synaptic Systems/400011	Mouse IgG	1:100
Human nuclei (hN)	Millipore / MAB4383	Mouse IgG1	1:100
Iba1	Wako/ 019-19741	Rabbit IgG	1:100
laminin	Sigma/ L9393	Rabbit IgG	1:30
MBP	Millipore / MAB386	Rat IgG	1:100
Ki67	Cell signaling / 9449	Mouse IgG	1:400
Ki67	Thermo Fisher Scientific / SP6	Rabbit IgG	1:200
OLIG2	Phosphosolutions 1538	Rabbit IgG	1:500
PDGFRα	Santacruze / sc-398206	Mouse IgG	1:200
PU.1	Thermo Fisher Scientific / phpu13	Mouse IgG1	1:50
P2RY12	Invitrogen / PA5-77671	Rabbit IgG	1:100
PSD95	Invitrogen 51-6900	Rabbit IgG	1:100
PSD95	Abcam / ab12093	Goat IgG	1:400
SPP1	Proteintech / 22952-1-AP	Rabbit IgG	1:200
Synapsin I	Millipore / AB1543P	Rabbit IgG	1:400
