# Supplementary material for: Human iPSC-derived mature microglia retain their identity and functionally integrate in the chimeric mouse brain
Source: Nat Commun. 2020 Mar 27;11:1577. doi: 10.1038/s41467-020-15411-9 (PMC7101330; doi:10.1038/s41467-020-15411-9)
Supplement: Supplementary file 3 — Description of Additional Supplementary Files [file 41467_2020_15411_MOESM3_ESM.docx]

Description of Additional Supplementary Files

**Title: Supplementary Data 1.** The top 10 enriched genes in each cluster. For each cell type cluster 7 identified by Seurat analysis, the top ten genes distinguishing that cluster from all other cells are shown, 8 as calculated by the FindAllMarkers function. The p-values (p_val) were calculated using a Wilcoxon 9 Rank Sum test, two-tailed, and were adjusted for multiple measurements using Benjamini-Hochberg 10 (p_val_adj). 11 12

**Title: Supplementary Data 2.** A list of differentially expressed genes between Xeno MG and mouse 13 microglia. Gene expression differences between human and mouse microglial clusters were calculated 14 by the Seurat FindMarkers function. The p-values (p_val) were calculated using a Wilcoxon Rank Sum 15 test, two-tailed, and were adjusted for multiple measurements using Benjamini-Hochberg (p_val_adj).
