# Supplementary material for: Human iPSC-derived mature microglia retain their identity and functionally integrate in the chimeric mouse brain
Source: Nat Commun. 2020 Mar 27;11:1577. doi: 10.1038/s41467-020-15411-9 (PMC7101330; doi:10.1038/s41467-020-15411-9)
Supplement: Supplementary file 6 — Reporting Summary [file 41467_2020_15411_MOESM6_ESM.pdf]

nature research

Corresponding author(s): Peng Jiang

Last updated by author(s): Feb 23, 2020

Reporting Summary

Nature Research wishes to improve the reproducibility of the work that we publish. This form provides structure for consistency and transparency in reporting. For further information on Nature Research policies, see Authors & Referees and the Editorial Policy Checklist .

Statistics

For all statistical analyses, confirm that the following items are present in the figure legend, table legend, main text, or Methods section.

n/a	Confirmed	
☐	☒	The exact sample size (n) for each experimental group/condition, given as a discrete number and unit of measurement
☐	☒	A statement on whether measurements were taken from distinct samples or whether the same sample was measured repeatedly
☐	☒	The statistical test(s) used AND whether they are one- or two-sided<br>*Only common tests should be described solely by name; describe more complex techniques in the Methods section.*
☐	☒	A description of all covariates tested
☐	☒	A description of any assumptions or corrections, such as tests of normality and adjustment for multiple comparisons
☐	☒	A full description of the statistical parameters including central tendency (e.g. means) or other basic estimates (e.g. regression coefficient) AND variation (e.g. standard deviation) or associated estimates of uncertainty (e.g. confidence intervals)
☐	☒	For null hypothesis testing, the test statistic (e.g. F, t, r) with confidence intervals, effect sizes, degrees of freedom and P value noted<br>*Give P values as exact values whenever suitable.*
☒	☐	For Bayesian analysis, information on the choice of priors and Markov chain Monte Carlo settings
☒	☐	For hierarchical and complex designs, identification of the appropriate level for tests and full reporting of outcomes
☐	☒	Estimates of effect sizes (e.g. Cohen's d, Pearson's r), indicating how they were calculated

Our web collection on statistics for biologists contains articles on many of the points above.

Software and code

Policy information about availability of computer code

Data collection	No custom code was used in this study. Single cell RNA Sequencing reads were aligned with pooled mouse (mm10) and human (hg19) reference genomes and the barcodes were interpreted using Cellranger software (10X Genomics, v. 3.0.0). The resulting matrices of gene counts x barcodes were coded by individual sample identifier and loaded into Seurat (v. 2.3.4) software 88-90 in R (version 3.6.1)/ Bioconductor 91. For analysis of human microglial sub-clusters, extracted human sample/barcode were restricted to human gene symbol results and re-analyzed with Seurat. Gene ontology analysis used the g:Profiler 95 website (version e98_eg45_p14_ce5b097) (https://biit.cs.ut.ee/gprofiler/gost).<br>For bulk RNA-sequencing analysis, total RNA was prepared with RNAeasy kit (QIAGEN) and libraries were constructed using 600 ng of total RNA from each sample and the TruSeqV2 kit from Illumina (Illumina, San Diego, CA) following manufacturers suggested protocol. The libraries were subjected to 75 bp paired read sequencing using a NextSeq500 Illumina sequencer to generate approximately 30 to 36 million paired-reads per sample. Fastq files were generated using the Bc12Fastq software, version 1.8.4. The genome sequence was then indexed using the rsem-prepare-reference command. Each fastq file was trimmed and checked for quality with fastp (v. 0.12.2), and then aligned to the UCSC hg38 human genome using Hisat2 (v.2.1.0). Transcript counts were extracted using the featureCounts function of the Rsubread package.<br>Confocal images were acquired using a LSM 710 Confocal microscope (Zeiss) and Zeiss Airyscan super-resolution microscope at 63X with 0.2 mm z-steps. Large scale images were obtained by confocal tile scan and automatically stitched using 10% overlap between tiles by Zen 2011 software (black edition, Zeiss).3D reconstructive images were processed by Zen 2011 software (black edition, Zeiss).To generate 3D-surface rendered images, super-resolution images were processed by Imaris software (Bitplane 9.5).To visualize the engulfed PSD95+ puncta within microglia, any fluorescence that was outside of the microglia was subtracted from the image by using the mask function in Imaris. Cell number and microglia process length and endpoints were counted with FIJI (ImageJ) software.
Data analysis	All data represent mean ± s.e.m. When only two independent groups were compared, significance was determined by two-tailed unpaired t-test with Welch's correction. When three or more groups were compared, one-way ANOVA with Bonferroni post hoc test or two-way ANOVA was used. A P value less than 0.05 was considered significant. The analyses were done in GraphPad Prism v.5.

For manuscripts utilizing custom algorithms or software that are central to the research but not yet described in published literature, software must be made available to editors/reviewers. We strongly encourage code deposition in a community repository (e.g. GitHub). See the Nature Research guidelines for submitting code & software for further information.

Data

Policy information about availability of data

All manuscripts must include a data availability statement. This statement should provide the following information, where applicable:

- Accession codes, unique identifiers, or web links for publicly available datasets
- A list of figures that have associated raw data
- A description of any restrictions on data availability

The accession numbers of the RNA sequencing data associated with Figure 4 and Figure 5 in this study are GEO: GSE129178 and GSE139161.
Raw sequencing reads from other publications were downloaded from GEO (series accessions GSE9907412, GSE9774417, GSE10233578, and GSE13343442).

Field-specific reporting

Please select the one below that is the best fit for your research. If you are not sure, read the appropriate sections before making your selection.

[x] Life sciences   [ ] Behavioural & social sciences   [ ] Ecological, evolutionary & environmental sciences

For a reference copy of the document with all sections, see nature.com/documents/nr-reporting-summary-flat.pdf

Life sciences study design

All studies must disclose on these points even when the disclosure is negative.

Sample size	No statistical methods were used to pre-determine sample sizes but our sample sizes are similar to those reported in previous publications.
Data exclusions	Data exclusion criteria were pre-established before the experiments. In order to analyze microglial morphology(Fig. 2 b-f), we exclude one transplanted mouse which had hydrocephalus and have abnormal microglial morphology.
Replication	At least 3 replicates were taken to verify the reproducibility of the experimental findings. All attempts at replication were successful.
Randomization	Animals were matched by age and similar numbers of male and female animals were used in nearly all comparisons. All treatments were given by a different researcher where possible to ensure blinding.
Blinding	The investigators were blind to group allocation during data collection and analysis.

Reporting for specific materials, systems and methods

We require information from authors about some types of materials, experimental systems and methods used in many studies. Here, indicate whether each material, system or method listed is relevant to your study. If you are not sure if a list item applies to your research, read the appropriate section before selecting a response.

Materials & experimental systems

n/a	Involved in the study	
[ ]	[x]	Antibodies
[ ]	[x]	Eukaryotic cell lines
[x]	[ ]	Palaeontology
[ ]	[x]	Animals and other organisms
[x]	[ ]	Human research participants
[x]	[ ]	Clinical data

Methods

n/a	Involved in the study	
[x]	[ ]	ChIP-seq
[x]	[ ]	Flow cytometry
[x]	[ ]	MRI-based neuroimaging

Antibodies

Antibodies used

CD163, R&D system / AF1067, Goat IgG, IF 1:200
CD235, Thermo Fisher Scientific / PA5-27154 ,Rabbit IgG, IF 1:100
CD43, Thermo Fisher Scientific / 14-0439-82, Mouse IgG1, 1:25
CD45, Thermo Fisher Scientific / 14-9457-82, Mouse IgG1, IF 1:40
CD68, Invitrogen/ MA5-13324, Mouse IgG1, 1:100
CD74 Invitrogen / JF40-10 Rabbit, IgG 1:200
hTMEM119, Thermo Fisher Scientific / PA-62505, Rabbit IgG, 1:400
Human nuclei (hN) Millipore / MAB4383, Mouse IgG1, 1:100
Iba1, Wako/ 019-19741, Rabbit IgG, 1:100

laminin, Sigma/ L9393, Rabbit IgG, 1:30
MBP Millipore / MAB386 Rat IgG, 1:100
Ki67, Cell signaling / 9449, Mouse IgG,1:400
Ki67, Thermo Fisher Scientific / SP6, Rabbit IgG, 1:200
mTMEM119, Synaptic Systems / 400 011, Mouse IgG, 1:100
OLIG2, Phosphosolutions 1538, Rabbit IgG, 1:500
PDGFRα Santacruze / sc-398206 Mouse IgG, 1:200
PU.1, Thermo Fisher Scientific / phpu13, Mouse IgG1, 1:50
PSD95, Invitrogen 51-6900, Rabbit IgG, 1:100
PSD95 Abcam / ab12093 Goat IgG, 1:400
SPP1 Proteintech / 22952-1-AP Rabbit IgG, 1:200
Synapsin I, Millipore / AB1543P, Rabbit IgG, 1:400

Validation

hCD163, Human CD163 Antibody, R&D Systems/AF 1607, polyclonal Goat IgG, 1: 100
https://www.rndsystems.com/products/human-cd163-antibody_af1607
Blood, 2007, June 15, 109: 5223. doi: 10.1182/blood-2006-08-036467

CD235, Thermo Fisher Scientific / PA-27154 ,Rabbit IgG, IF 1:100
https://www.thermofisher.com/antibody/product/CD235a-Antibody-Polyclonal/PA5-27154
No information

CD43, Thermo Fisher Scientific / 14-0439-82, Mouse IgG1, 1:25
https://www.thermofisher.com/antibody/product/CD43-Antibody-clone-eBio84-3C1-84-3C1-Monoclonal/14-0439-82
Stem Cells Transl Med. 2018 Jan;7(1):34-44. doi: 10.1002/sctm.17-0021. Epub 2017 Nov 21.

CD45, Thermo Fisher Scientific / 14-9457-82, Mouse IgG1, IF 1:40
https://www.thermofisher.com/antibody/product/CD45-Antibody-clone-CD45-2B11-Monoclonal/14-9457-82
JCI Insight. 2016 Jul 21;1(11):e87680. doi: 10.1172/jci.insight.87680.

CD68, Invitrogen/ MA5-13324, Mouse IgG1, 1:100
https://www.thermofisher.com/antibody/product/CD68-Antibody-clone-KP1-Monoclonal/MA5-13324
Redox Biol. 2018 May;15:22-33. doi: 10.1016/j.redox.2017.11.013. Epub 2017 Nov 16.

hTMEM119, Thermo Fisher Scientific / PA-62505, Rabbit IgG, 1:400
https://www.thermofisher.com/antibody/product/TM119-Antibody-Polyclonal/PA5-62505
No information

CD74, Invitrogen/MA5-32529, recombinant rabbit monoclonal IgG antibody, 1: 200
https://www.thermofisher.com/antibody/product/CD74-Antibody-clone-JF40-10-Recombinant-Monoclonal/MA5-32529
No information

Human nuclei (hN) Millipore / MAB4383, Mouse IgG1, 1:100
http://www.emdmillipore.com/US/en/product/Anti-Nuclei-Antibody-clone-3E1.3,MM_NF-MAB4383
J Clin Med. 2015 Jan 14;4(1):159-71. doi: 10.3390/jcm4010159.

Iba1, Wako/ 019-19741, Rabbit IgG, 1:100
http://www.e-reagent.com/uh/Shs.do?dspWkfcode=019-19741
J Neurosci. 2012 Aug 8;32(32):10809-18. doi: 10.1523/JNEUROSCI.1487-12.2012.

laminin, Sigma/ L9393, Rabbit IgG, 1:30
https://www.sigmaaldrich.com/catalog/product/sigma/l9393?lang=en&region=US
Am J Pathol. 2002 Aug;161(2):727-35.

MBP: Anti-Myelin Basic Protein Antibody, Millipore Sigma/AB5864, polyclonal Rabbit IgG, 1:100
https://www.citeab.com/antibodies/223919-ab5864-anti-myelin-basic-protein-antibody
Nat Neurosci. 2013 Sep;16(9):1211-1218. doi: 10.1038/nn.3469. Epub 2013 Jul 21.

Ki67, Cell signaling / 9449, Mouse IgG,1:400
https://www.cellsignal.com/products/primary-antibodies/ki-67-8d5-mouse-mab/9449
Oncol Lett. 2019 May;17(5):4255-4262. doi: 10.3892/ol.2019.10090. Epub 2019 Feb 28.

Ki67, Thermo Fisher Scientific / SP6, Rabbit IgG, 1:200
https://www.thermofisher.com/antibody/product/Ki-67-Antibody-clone-SP6-Monoclonal/MA5-14520

Nat Commun. 2018 Aug 24;9(1):3425. doi: 10.1038/s41467-018-05726-z.

mTMEM119, Synaptic Systems / 400 011, Mouse IgG, 1:100
https://www.sysy.com/products/tmem119/facts-400011.php
Proc Natl Acad Sci U S A. 2016 Mar 22;113(12):E1738-46. doi: 10.1073/pnas.1525528113. Epub 2016 Feb 16.

OLIG2, Phosphosolutions 1538, Rabbit IgG, 1:500
https://www.phosphosolutions.com/shop/olig2-antibody/
PLoS One. 2017 Nov 2;12(11):e0187530. doi: 10.1371/journal.pone.0187530. eCollection 2017.

PPDGFRα, Santa Cruz/sc-398206, Mouse IgG, 1:200
https://datasheets.scbt.com/sc-398206.pdf
Cell. 2018. 174: 1158-1171.e19. DOI: 10.1016/j.cell.2018.06.028

PU.1, Thermo Fisher Scientific / phpu13, Mouse IgG1, 1:50
https://www.thermofisher.com/antibody/product/PU-1-Antibody-clone-phpu13-Monoclonal/14-9819-82
No information

PSD95, Invitrogen 51-6900, Rabbit IgG, 1:100
https://www.thermofisher.com/antibody/product/PSD-95-Antibody-Polyclonal/51-6900
Sci Rep. 2016 May 16;6:26060. doi: 10.1038/srep26060.

PSD95, Abcam/ab12093, Goat IgG, 1:400
https://www.abcam.com/psd95-antibody-ab12093.html
J Neurosci, 2018 Sep 12, 38 (37), 7935-7951, DOI: 10.1523/JNEUROSCI.0801-18.2018

SPP1, secreted phosphoprotein 1/Osteopontin Antibody, Proteintech/22952-1-AP, Rabbit IgG, 1:200
https://www.ptgcn.com/products/OPN,-SPP1-Antibody-22952-1-AP.htm
Stem Cells Int, 2015, 565732, DOI: 10.1155/2015/565732

Synapsin I, Millipore / AB1543P, Rabbit IgG, 1:400
http://www.emdmillipore.com/US/en/product/Anti-Synapsin-I-Antibody,MM_NF-AB1543P
J Cell Sci. 2015 Mar 15;128(6):1241-52. doi: 10.1242/jcs.167544. Epub 2015 Jan 27

Eukaryotic cell lines

Policy information about cell lines

Cell line source(s)	One healthy control hiPSC line and H9 ESC line were used this study. The hiPSC line were generated from healthy person-derived fibroblasts using the "Yamanaka" reprogramming factors, as reported in our previous study. H9 ESC was provided from WiCell Research Institute.
Authentication	The hiPSC line and H9 ESC line have been fully characterized by performing karyotyping, teratoma assay, DNA fingerprinting STR (short tandem repeat) analysis, gene expression profiling, and Pluritest (www.PluriTest.org), a robust open-access bioinformatic assay of pluripotency in human cells based on their gene expression profiles, as described in our previous study.
Mycoplasma contamination	All cell lines tested negative for mycoplasma contamination.
Commonly misidentified lines (See ICLAC register)	No cell line used in this paper are listed in the database of commonly misidentified cell lines maintained by ICLAC.

Animals and other organisms

Policy information about studies involving animals; ARRIVE guidelines recommended for reporting animal research

Laboratory animals	Neonatal Rag2-/-hCSF1 immunodeficient mice (C;129S4-Rag2tm1.1Flv Csf1tm1(CSF1)Flv Il2rgtm1.1Flv/J, The Jackson Laboratory) were transplanted with the donor-derived cells at without gender bias. These mice were kept up to 6 months before they were tested for the engraftment of human cells and other experiments.
Wild animals	The study did not involve wild animals.
Field-collected samples	The study did not involve field-collected samples.
Ethics oversight	All animal work was performed without gender bias under the Institutional Animal Care and Use Committee (IACUC) protocol approved by Rutgers University IACUC Committee.

Note that full information on the approval of the study protocol must also be provided in the manuscript.
